# Oncological outcomes of patients with inflammatory bowel disease undergoing segmental colonic resection for colorectal cancer and dysplasia: systematic review

**DOI:** 10.1093/bjsopen/zrae052

**Published:** 2024-05-31

**Authors:** Amira Shamsiddinova, Jennie Burch, Mohammed Deputy, Christopher Rao, Guy Worley, Harry Dean, Siwan Thomas-Gibson, Omar Faiz

**Affiliations:** Department of Surgery and Gastroenterology, St Mark’s Hospital and Academic Institute, London, UK; Department of Surgery and Cancer, Imperial College London, London, UK; Department of Surgery and Gastroenterology, St Mark’s Hospital and Academic Institute, London, UK; Department of Surgery and Gastroenterology, St Mark’s Hospital and Academic Institute, London, UK; Department of Surgery and Cancer, Imperial College London, London, UK; Department of Surgery and Cancer, Imperial College London, London, UK; Department of Colorectal Surgery, North Cumbria Integrated Care NHS Foundation Trust, Cumberland Infirmary, Carlisle, UK; Department of Surgery and Gastroenterology, St Mark’s Hospital and Academic Institute, London, UK; Department of Surgery and Cancer, Imperial College London, London, UK; Department of Surgery and Gastroenterology, St Mark’s Hospital and Academic Institute, London, UK; Department of Surgery and Cancer, Imperial College London, London, UK; Department of Surgery and Gastroenterology, St Mark’s Hospital and Academic Institute, London, UK; Department of Metabolism, Digestion and Reproduction, Imperial College London, London, UK; Department of Surgery and Gastroenterology, St Mark’s Hospital and Academic Institute, London, UK; Department of Surgery and Cancer, Imperial College London, London, UK

## Introduction

Dysplasia in inflammatory bowel disease (IBD) is a premalignant precursor to colorectal cancer (CRC); however, literature reporting progression rates is of low quality^1^. International guidelines on neoplasia management recommend proctocolectomy for CRC, endoscopically unresectable dysplasia, non-visible high-grade dysplasia (HGD), and multifocal low-grade dysplasia^2^, with a recent trend towards segmental colorectal resections, evidenced by population studies reporting 40–70% of patients with IBD-associated CRC undergoing segmental colectomy^3–5^. The aim of this systematic review was to evaluate surgical and oncological outcomes of segmental colectomy compared with proctocolectomy for neoplasia in IBD to inform decision-making.

## Methods

This systematic review was registered on 22 December 2021 in PROSPERO, the international prospective register of systematic reviews, with registration number CRD42021292891, and was conducted in accordance with the PRISMA guidelines (PRISMA checklist is available as *Supplementary material*)^6^. This work did not require ethical approval. Relevant databases and grey literature were searched using a search strategy (*Supplementary Methods*). COVIDENCE software was used for screening and data extraction, independently by two authors. Studies were included if they were in English, were published between 1990 and 2023, reported on IBD patients who underwent segmental colectomy for dysplasia or cancer, and reported oncological or postoperative outcomes. Systematic reviews and case reports were excluded. Study quality was assessed^7^. The primary outcomes for the review were oncological.

## Results

**Fig. 1:**
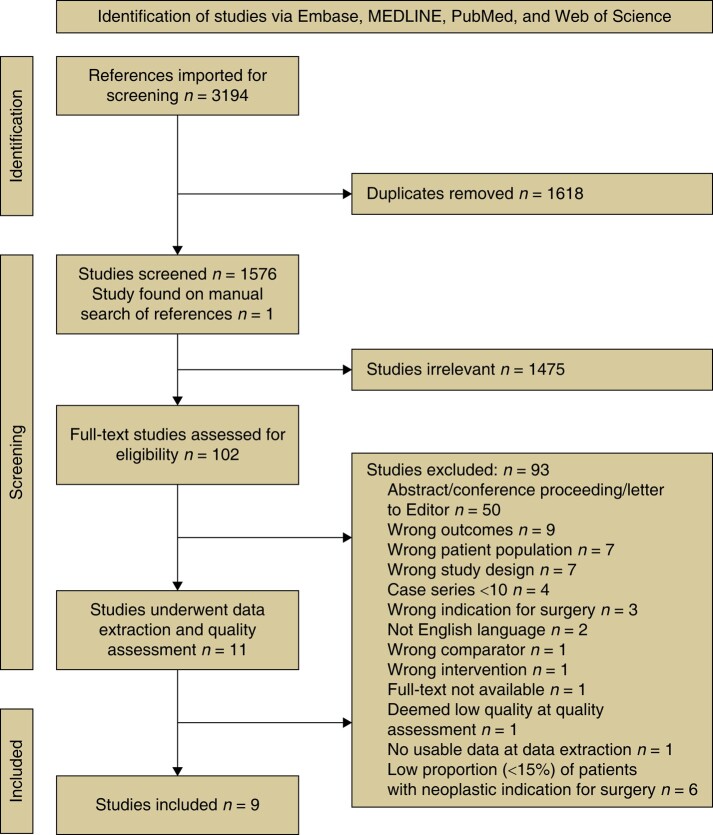
PRISMA flow diagram for the systematic review

Initial searches returned 3194 results; nine studies^5,8–15^ were included (*Fig. 1*). All studies were retrospective and observational, including 4181 patients with outcomes of 3810 segmental resections and 371 proctocolectomies. A total of four studies^8,9,11,13^ compared segmental colectomy with proctocolectomy outcomes. Male patients and those with ulcerative colitis were over-represented (greater than 60%). There was significant study heterogeneity (33 of 95 fields ‘no/unclear’ in quality assessment; see the *Supplementary Results*), including indication for surgery. The studies spanned 1977 to 2020. A total of three studies^11,13,15^ reported on ulcerative colitis, one study^14^ reported on Crohn’s disease, and most studies included all IBD subtypes (*Tables S1*, *S2*).

Studies reported low rates of missed synchronous cancers (0.5–4.5%) and HGD (1.3–5.8%)^5,12–14^. Rates of metachronous cancers and dysplastic lesions were 0–33% at a median of 1.58–3 years^9–15^, with two studies reporting cumulative CRC incidence rates of 6.5% (95% c.i. 1.1% to 19.2%) at 3 and 5 years^12^ and 1.3% at 3 years, increasing to 6.3% at 15 years^5^. The studies reporting the highest metachronous CRC rates (29%^14^ and 33%^9^) were based on only six patients undergoing proctectomy^9^ or had high recurrence rates within the first year at the anastomosis, raising the possibility of inadequate preoperative surveillance and incomplete resection margins^14^. Metastasis during follow-up included lung metastasis and liver metastasis, and was similar in both groups^11,15^ (*Table 1* and *Table S3*). Surgical outcomes included a similar duration of hospital stay^11,15^, a similar postoperative complication rate^11,15^, and a similar hospital readmission rate^11^ for both segmental resection and proctocolectomy. Subsequent surgery included completion surgery and further segmental resections^10,13,15^ for refractory colitis, dysplasia, cancer, and stenosis. A total of four studies^10,11,13,15^ reported on colitis relapse during follow-up (up to 84%), often necessitating medical therapy. Mortality within 30 days occurred after emergency procedures^15^, not planned operations^11^, or was similar in both groups^12^ (*Tables S4–S6*). Overall survival was similar for segmental resection and proctocolectomy for CRC^8,9,11^ (*Table 1*).

**Table 1:** Summary of oncological outcomes from the nine studies included in the systematic review

Study	Synchronous cancer	Metachronous cancer rate	Overall survival
Derks *et al*.^12^ 2023 (HGD and cancer cohort)	1 of 172 (0.5%)	7 of 139* (5%) at a median of 2.3 (i.q.r. 1.2–3.8) years Cumulative HGD and cancer rate: Subtotal colectomy (*n* = 45): 5.6% (95% c.i. 1%, 16.7%) at 3 and 5 years Other segmental resection (*n* = 56): 2.7% (95% c.i. 0.2%, 12.4%) at 1 year 6.5% (95% c.i. 1.1%, 19.2%) at 3 and 5 years	No significant difference in all-cause mortality at a median of 70 months
Birch *et al*.^5^ 2022 (cancer-only cohort)	166 of 5141 (3.2%)	Cumulative cancer rate: Subtotal colectomy: 1% within 3 years Other segmental resection: 1.3% within 3 years 6.3% within 15 years	–
Bogach *et al*.^8^ 2021 (cancer-only cohort)	–	–	Equivalent in proctocolectomy and segmental resection (HR 0.99 (95% c.i. 0.78, 1.27))
Frontali *et al*.^15^ 2020 (benign, dysplasia, and cancer cohort)	–	3 of 72 (4%) at a median of 1.58 (range 0.2–13.2) years	–
Krugliak Cleveland *et al*.^10^ 2019 (benign, dysplasia, and cancer cohort)	–	0 of 17 (0%) at a median of 1.4 (range 0.3–19.0) years	–
Khan *et al*.^11^ 2017 (cancer-only cohort)	–	0 of 25 (0%) at a median of 9 years	Worse in the segmental resection cohort than in the proctocolectomy cohort—due to preoperative patient characteristics
Klos *et al*.^9^ 2016 (cancer-only cohort)	–	2 of 6 (33%) at <2 years	Equivalent in proctectomy and proctocolectomy; disease-free survival at 3 years was significantly lower in the proctectomy group compared with the proctocolectomy group
Maser *et al*.^14^ 2013 (dysplasia and cancer cohort)	3 of 75 (4%)	22 of 75 (29%) at a median of 3 (range 1–38) years† Subtotal colectomy *versus* segmental resection: not statistically significant	–
Lindberg *et al*.^13^ 2006 (dysplasia and cancer cohort)	1 of 22 (4.5%)	0 of 22 (0%) at unknown time point	–

*Segmental, subtotal colectomy, and endoscopic resection patients. †Cancers occurring >6 months after surgery; 8 of 22 occurred in the first year. HGD, high-grade dysplasia; i.q.r., interquartile range.

## Discussion

This systematic review consolidates evidence on oncological and surgical outcomes after segmental colorectal resection for colorectal dysplasia and cancer in the setting of IBD. Inter-study heterogeneity precluded comparisons using meta-analysis. However, it is possible to conclude that rates of synchronous and metachronous cancers are low, rates of postoperative colitis flares requiring medical therapy are considerable, and overall survival is not impacted by surgery type. The latter is also supported by findings of other studies^4,16^, excluded here due to differences in design and methodology.

Findings of synchronous cancer rates of 0.5–4.5% are consistent with the reported 1–14% (depending on grade of dysplasia) in another systematic review^1^ comparing the histology of resected colectomy specimens with preoperative endoscopic findings and are similar to the findings of other studies when the indication was CRC^17,18^. The term ‘missed synchronous cancer’ in the literature assumes the lesion was missed during preoperative endoscopy and found at colectomy and most studies in the present systematic review did not report this.

One of the included studies^5^ suggested that the cumulative metachronous CRC incidence rate in patients operated on for IBD-associated CRC in England was 1.3% within 3 years of segmental resection, increasing to 6.3% within 15 years, whereas another^12^ showed that patients operated on for HGD and CRC in the Netherlands had cumulative CRC rates of 2.7% at 1 year, rising to 6.5% at 3 years. Other previously published studies not included here reported higher metachronous cancer recurrence rates (17.2%^4^ and 9%^16^); however, in the Gearhart *et al*.^4^ study the patients were greater than 65 years old and in Kiran *et al*.^16^ only 11 patients underwent segmental resections, which may account for the higher rates. The study^14^ that reported the highest rate of metachronous cancer defined all neoplasia discovered after surgery as ‘metachronous’, and applying the authors’ definition to this study by Maser *et al.* resulted in fewer metachronous cancers (from 39% to 29%). It is also possible that the patients in the Maser *et al.* study had incompletely resected cancers and had inadequate endoscopic surveillance perioperatively.

Other studies^5,12^ reported similar findings for rectal cancer risk in those undergoing subtotal colectomy to another systematic review^19^ with prior CRC identified as a major risk factor for development of rectal neoplasia. However, this systematic review by Derikx *et al.* included studies with all indications for resection, as well as older studies, and therefore their cohort is inherently different to the current IBD population with improved surveillance methods and medical management of IBD^1^. Metachronous cancer/HGD risk already reported in the literature^1^ is not a reliable surrogate, as a larger segment is removed during surgery than endoscopic resection, which may reduce the area affected by field cancerization and thus reduce the risk of local recurrence. Derks *et al*.^12^ did indeed find a higher rate of metachronous advanced neoplasia recurrence in patients undergoing endoscopic resection compared with surgery. The available recurrence data in the present study, however, seem to demonstrate that, when there is a neoplastic recurrence, it is mostly localized and at an early stage, which may be attributable to heightened endoscopic vigilance. This is consistent with the authors’ conclusion that overall survival is not affected by the type of surgery, as the risks of metachronous and synchronous cancers are mitigated by timely management. Thus, the current practice of offering proctocolectomy to IBD patients with unifocal dysplasia/cancer is therapeutic for the neoplastic segment and prophylactic for the remainder of the colorectum.

The limitations of this study are that the included studies were retrospective, highly heterogeneous, and mostly from single institutions. Outcomes are reported for small patient cohorts, due to relatively low incidences of IBD-associated dysplasia and the lower incidence of segmental resection for this indication. Also, studies were carried out at different institutions, introducing heterogeneity through different practices. Furthermore, older studies would now be outdated, due to improved endoscopic dysplasia detection, standardized nomenclature and surveillance, and use of biologicals^1^.

Despite these limitations, data suggest that synchronous and metachronous cancer rates are low, certainly lower than the threshold for patients considering colectomy^20^. Findings from national population-based studies demonstrate the clear popularity of segmental resection over proctocolectomy^3–5,8^ and the willingness of patients to accept risks in favour of a less radical alternative. Prospective studies are needed to determine the safety and efficacy of segmental resection for IBD-associated neoplasia and enable informed consent within a patient-centred shared decision-making context.

## Supplementary Material

zrae052_Supplementary_Data

## Data Availability

The template data collection forms and data extracted from included studies can be obtained by e-mailing the corresponding author.
