# Supplementary material for: Oncological outcomes of patients with inflammatory bowel disease undergoing segmental colonic resection for colorectal cancer and dysplasia: systematic review
Source: BJS Open. 2024 May 31;8(3):zrae052. doi: 10.1093/bjsopen/zrae052 (PMC11143477; doi:10.1093/bjsopen/zrae052)
Supplement: zrae052_Supplementary_Data [file zrae052_supplementary_data.zip › Supplementary_Materials.docx]

Systematic review: Oncological outcome of patients with Inflammatory Bowel Disease undergoing segmental colonic resection for colorectal cancer and dysplasia

Amira Shamsiddinova MRCS, BSc^1,2^, Jennie Burch RN, MSc^1^, Mohammed Deputy MRCS, BSc ^1,2^, Christopher Rao FRCS, PhD^2,3^, Guy Worley FRCS, PhD^1,2^, Harry Dean MRCS, FHEA^1,2^, Siwan Thomas-Gibson FRCP, MD^1,2^, Omar Faiz FRCS, MD^1,2^

**Institutions:**

^1.^ St Mark’s Academic Institute, Acton Lane, London NW10 7NS, United Kingdom

^2.^ Imperial College London, Exhibition Road, London SW7 2BX, United Kingdom

^3.^ North Cumbria Integrated Care NHS Foundation Trust, Cumberland Infirmary, Infirmary Street,

Carlisle CA2 7HY, United Kingdom

Authors

**Corresponding author:**

Amira Shamsiddinova

St Mark’s Academic Institute

Central Middlesex Hospital

Acton Lane

London, United Kingdom

NW10 7NS

Email: as3305@ic.ac.uk

ORCID ID: 0000-0002-9108-4507

**Supplementary Materials - Index**

| **Supplementary Methods** |  |
| --- | --- |
| Search Strategy | *page 3* |
| Definitions | *Page 3* |
| Data Extraction | *Page 3* |
| **Supplementary Results** |  |
| Quality Assessment | *page 4* |
| **Supplementary Figures and Tables** |  |
| Supplementary Table 1. Summary of studies | *Page 6* |
| Supplementary Table 2. Patient demographics | *Page 8* |
| Supplementary Table 3. Metachronous neoplasia | *Page 9* |
| Supplementary Table 4. Surgical outcomes | *Page 10* |
| Supplementary Table 5. Mortality and causes | *page 11* |
| Supplementary Table 6. IBD flare and Pouchitis incidence post-surgery at end of follow-up | *Page 12* |
| **References** | *Page 13* |

**Supplementary Methods**

Search strategy used:

1. ulcerative colitis.mp. or exp ulcerative colitis/

2. crohn's disease.mp. or exp Crohn disease/

3. inflammatory bowel disease.mp. or inflammatory bowel disease/

4. 1 or 2 or 3

5. exp colon tumor/

6. ((colorectal or colon or colonic or colitis or sigmoid or rectal or rectum) adj3 (neoplas* or cancer* or malignan* or polyp* or tumo?r*)).mp. [mp=ti, ab, tx, ct, ot, nm, hw, fx, kf, ox, px, rx, ui, sy]

7. exp dysplasia/

8. dysplas*.mp. [mp=ti, ab, tx, ct, ot, nm, hw, fx, kf, ox, px, rx, ui, sy]

9. exp hemicolectomy/ or exp sigmoidectomy/ or exp segmentectomy/

10. (hemicolectom* or (hemi adj1 colectom*) or sigmoidectom* or segmentectom*).mp. [mp=ti, ab, tx, ct, ot, nm, hw, fx, kf, ox, px, rx, ui, sy]

11. ((segmental or subtotal or "sub total" or total or partial or limited) adj3 (colectom* or proctectom* or resection* or excision)).mp. [mp=ti, ab, tx, ct, ot, nm, hw, fx, kf, ox, px, rx, ui, sy]

12. 9 or 10 or 11

13. 5 or 6 or 7 or 8

14. 4 and 12 and 13

15. limit 14 to (english language and yr="1990 -Current")

**Definitions.** Segmental resection was defined as the removal of any segment of the colon or rectum resulting in a remnant of colon or rectum left in situ. This included Right hemicolectomy, Left hemicolectomy, sigmoidectomy, anterior resection of rectum, proctectomy/abdominoperineal resection, and subtotal colectomy and ileorectal anastomosis (IRA). The comparator, Proctocolectomy was defined as removal of the whole colon and rectum, with resultant Ileal-Pouch Anal Anastomosis (restorative proctocolectomy and ileal-pouch anal-anastomosis - IPAA) or end-ileostomy.

**Data Extraction.** Data was collected by two authors independently, and included first author, title of article, year of publication, country in which the study was conducted, aim of the study, study design, start and end dates of the study, declared conflicts of interest, funding sources, type of IBD, inclusion and exclusion criteria, total number of participants in the study, total number of patients who had segmental resections, total who had proctocolectomy, demographic data (age, gender, comorbidities), disease-specific data (duration, severity, extension, age at diagnosis) for the populations, acuity of operation, length of follow-up, indication and frequency of surgery, mortality and outcome data specified below. Discrepancies and conflicts were resolved upon discussion between the authors. In cases where the data was not available in the published paper, the corresponding authors were contacted to retrieve the required missing data points, as appropriate, otherwise this data was not included in analysis.

**Supplementary Results**

Quality Assessment results of the studies that passed the data extraction stage. *Source:* JBI Critical Appraisal Checklist for Cohort Studies (*Critical Appraisal Tools | JBI*, n.d.). NA=Not applicable

| **Study ID** | **Q1** | **Q2** | **Q3** | **Q4 Q5** | | **Q6** | **Q7** | **Q8** | **Q9** | **Q10** | **Q11** | **Overall appraisal** |
| --- | --- | --- | --- | --- | --- | --- | --- | --- | --- | --- | --- | --- |
| Sensi 2021 | no | yes | yes | yes | yes | NA | no | yes | yes | NA | yes | Include |
| Derks 2023 | no | yes | yes | yes | yes | yes | yes | yes | yes | NA | yes | Include |
| Bogach 2021 | no | yes | yes | yes | yes | NA | yes | yes | yes | NA | yes | Include |
| Frontali 2020 | NA | yes | yes | yes | unclear | NA | unclear | no | unclear | no | yes | Include |
| Khan 2017 | no | yes | yes | yes | yes | NA | yes | yes | yes | no | unclear | Include |
| Krugliak-Cleveland 2019 | NA | yes | yes | yes | no | NA | yes | no | unclear | no | no | Include |
| Lindberg 2006 | unclear | yes | yes | unclear | no | NA | yes | no | no | no | unclear | Include |
| Maser 2013 | NA | yes | yes | yes | unclear | unclear | unclear | yes | unclear | no | no | Include |
| Klos 2016 | yes | yes | yes | yes | yes | yes | no | yes | no | no | yes | Include |
| Birch 2022 | NA | yes | yes | yes | yes | yes | yes | yes | NA | NA | yes | Include |
| Sugita 2021 | no | yes | unclear | unclear | unclear | unclear | no | no | no | no | yes | Exclude |

| **Quality Assessment Questions** | |
| --- | --- |
| 1 | Were the two groups similar and recruited from the same population? |
| 2 | Were the exposures measured similarly to assign people to both exposed and unexposed groups? |
| 3 | Was the exposure measured in a valid and reliable way? Was segmental resection defined? |
| 4 | Were confounding factors identified? |
| 5 | Were strategies to deal with confounding factors stated? |
| 6 | Were the groups/participants free of the outcome at the start of the study (or at the moment of exposure)? |
| 7 | Were the outcomes measured in a valid and reliable way? Surveillance method/time frame stated |
| 8 | Was the follow-up time reported and sufficient to be long enough for outcomes to occur? |
| 9 | Was follow-up complete, and if not, were the reasons to loss to follow-up described and explored? |
| 10 | Were strategies to address incomplete follow-up utilized? |
| 11 | Was appropriate statistical analysis used? |

**Supplementary Figures and Tables**

| **Study ID** | **Title** | **Country** | **Aim of study** | **Study period** | **IBD Type** | **Total**  **(SR/PC)** | **Length of Follow-up** |
| --- | --- | --- | --- | --- | --- | --- | --- |
| (Bogach et al., 2021) | Extent of Surgical Resection in Inflammatory Bowel Disease-Associated Colorectal Cancer: a Population-Based Study | Canada | Describe, at a population level, the type of surgical resections that are being performed for patients with IBD-associated CRC in the province of Ontario and to examine the relationship between extent of surgical resection and survival. The secondary purpose is to determine if type of surgery performed is associated with the differences in survival seen in CD compared to UC | 2007-2015 | All IBD | 965  (678/287) | Median 5.9 years |
| (Klos et al., 2016) | Impaired outcome colitis-associated rectal cancer versus sporadic cancer | United States- Washington University | To compare oncologic outcomes in patients with colitis-associated rectal cancer (CARC) to a matched (1:2) group of patients undergoing resection for sporadic rectal cancer | 1993-2012 | All IBD | 27  (6/21) | CARC Median 2.7 years (IQR 1.3 -10.1) |
| (Birch et al., 2022) | Inflammatory Bowel Disease-Associated Colorectal  Cancer Epidemiology and Outcomes: An English  Population-Based Study | England | To examine the characteristics, surgical treatment, and outcomes for Patients with IBD-CRC within the English National Health Service (NHS). | 2005-2018 | All IBD | 5141  (2858/-) | NA |
| (Frontali et al., 2020) | Segmental Colectomy for Ulcerative Colitis: Is There a Place in Selected Patients Without Active Colitis? An International Multicentric Retrospective Study in 72 Patients | France, Italy, Sweden, Spain, USA | To report a multicentric experience of segmental colectomy (Right, sigmoid, left, proctectomy) [SC] in UC patients without active colitis, to assess if SC can or cannot represent an alternative to ileal pouch-anal anastomosis (IPAA) | not stated | UC | 72  (72/-) | Median 40 months (range 1-600 months) |
| (Maser et al., 2013) | High Rates of Metachronous Colon Cancer or Dysplasia After Segmental Resection or Subtotal Colectomy in Crohn’s Colitis | United States- Mount Sinai NY | To determine the risk of metachronous colon cancer or dysplasia after segmental resection or subtotal colectomy (STC) for HGD or cancer in patients with colorectal cancer | 2001-2011 | CD | 75  (75/-) | Mean 5.3 years; median 2.9 years (range 0-38yrs) |
| (Krugliak Cleveland et al., 2019) | Efficacy and Follow-up of Segmental or Subtotal Colectomy in Patients With Colitis-Associated Neoplasia | United States, Chicago | To report our experience and follow-up evaluation of segmental resections for preoperative neoplasia in patients with CD or UC | not stated | All IBD | 17  (17/-) | Median 17 months (range 3-228) |
| (Khan et al., 2017) | Segmental resection is a safe oncological alternative to total proctocolectomy in elderly patients with ulcerative colitis and malignancy | United States- Veterans Affairs Healthcare system | To evaluate oncological, as well as surgical and disease-specific, outcomes after segmental resection in UC for colorectal cancer | 2001-2011 | UC | 59  (25/34) | Median 9 years |
| (Lindberg et al., 2006) | Surgery for neoplastic changes in ulcerative colitis: can limited resection be justified? Outcome for patients who underwent limited surgery | Sweden | Reporting from an ongoing surveillance programme of unselected patients with UC from a defined catchment area, focusing on the outcome for the patients who underwent limited surgery, with special attention to those with neoplastic changes | 1977-2003 | UC | 51  (22/29) | NA |
| (Derks et al., 2023)* | Endoscopic and Surgical Treatment Outcomes of Colitis-Associated Advanced Colorectal Neoplasia: A Multicentre Cohort Study | The Netherlands | To compare cumulative incidences of synchronous and metachronous colorectal neoplasia as well as mortality following advanced neoplasia in CD  and UC patients who underwent proctocolectomy, (sub)total colectomy, partial colectomy or  endoscopic resection, and to determine factors associated with advanced neoplasia  treatment choice. | 1991-2020 | All IBD | 189  (56/33*) | Median 27 months (IQR 7-69) |
| (Sensi et al., 2022)^*^ | Long-term Oncological Outcome of Segmental Versus Extended Colectomy for Colorectal Cancer in Crohn’s Disease: Results from an International Multicentre Study | Italy, England, USA, France Spain | To compare the effects of segmental colectomy and extended colectomy on long-term prognosis of patients with CRC arising in the background of CD | 2010-2020 | CD | 99  (66/33*) | Median 43 months (range 31-62) |

**Supplementary Table 1.** Summary of studies. *Sensi et al., 2021 and Derks et al., 2023 excluded subtotal colectomy from their definition of segmental resection, instead considering it with proctocolectomy as ‘extended resection’. Therefore, their results cannot be directly compared. Sensi et al., 2021 also included pelvic exenteration in ‘segmental resection’, therefore this study was excluded from the review. SR= segmental resection; PC= Proctocolectomy; IRA= ileorectal anastomosis; IPAA=ileal-pouch-anal anastomosis; DR=diverted rectum; CD=Crohn’s Disease; UC=Ulcerative Colitis; IBD=Inflammatory Bowel Disease; NA=Not Available.

| **^Study ID^** | **^Males,^ *^n^* ^(%)^** | **^Age at colectomy (years)^** | **^IBD subtype,^ *^n^* ^(%)^** | **^Age at IBD diagnosis^** | **^Disease duration (years)^** | **^Disease extension^** | **^Disease severity^** | **^Indication for surgery,^ *^n^* ^(%)^** | **^Surgery type^** |
| --- | --- | --- | --- | --- | --- | --- | --- | --- | --- |
| (Derks et al., 2023) | SR: 32 (57) | SR: Median 62 (IQR 51.0–70.0) | CD: 25 (45)  UC: 28 (50)  IBD-U: 2 (4) | SR: Median 34 (IQR 4.0–52.5) | SR: Median 19.0 (IQR 13.0–31.0) | SR: Montreal 1- 2  Montreal 2- 13  Montreal 3- 15  CD: Colon <50%- 12  Colon >50%- 14 | NA | SR: (HGD) Dysplasia – 12 (21)  Cancer – 44 (79) | ‘Segment resection’ – 29  Right hemi – 14  Ileocaecal resection – 3  Left hemi – 3 |
| (Birch et al., 2022) | Total cohort: 3091 (60) | Median 66 (IQR 54 – 76) | CD: 1512 (29)  UC: 3123 (61)  IBD-U: 354 (7)  IBD & Cholangitis: 152 (3) | NA | NA | NA | NA | Dysplasia – NA  Cancer – 2858 (100) | Any other SR – 2357  Subtotal colectomy – 501 |
| (Bogach et al., 2021) | SR: 394 (58)  PC: 180 (63) | SR: Median 71 (61-79)  PC: Median 66 (57-75) | CD: 366 (38)  UC: 599 (62) | NA | NA | NA | NA | SR – Dysplasia – NA  Cancer – 678 (100)  PC – Dysplasia – NA  Cancer – 287 (100) | Any other SR – 522  Subtotal colectomy – 156  PC – 287 |
| (Frontali et al., 2020) | 50 (70) | Mean 57 (SD 17, range 14 - 84) | CD: 0  UC: 72 (100) | Mean 46 (SD 18, range 11-86) | NA | Ileal only: 0  Proctitis: 12  Left-sided: 31  Right sided: 15  Unk: 14 | Mayo 0-7  mayo 1-21  mayo 2-14  mayo 3-7  Unk -23 | Dysplasia – 8 (11)  Cancer – 27 (38) | Right hemi – 24  Left hemi – 9  Sigmoidectomy – 28  Proctectomy – 11  Subtotal colectomy – 0 |
| (Krugliak Cleveland et al., 2019) | NA | Median 64 (range 40 – 78) | CD: 11 (65)  UC: 6 (35) | NA | Median 20.5 (range 5–46) | NA | NA | Dysplasia – 14 (82)  Cancer – 3 (18) | ‘Segmental colectomy’ – 12  Subtotal colectomy/IRA – 5 |
| (Khan et al., 2017) | SR: 25 (100)  PC: 34 (100) | SR: Median 73  PC: Median 61.7 | CD: 0  UC: 59 (100) | SR: Median 62  PC: Median 38 | SR: Median 7  PC: Median 19.5 | SR/PC:  Montreal 0- 15/8  Montreal 1- 3/1  Montreal 2- 3/14  Montreal 3- 4/11 | SR/PC:  Froslie 0 – 15/8  Froslie 1 – 4/12  Froslie 2- 6/14 | SR: Dysplasia - NA  Cancer – 25 (100)  PC: Dysplasia – NA  Cancer – 34 (100) | Right hemi – 10  Left hemi – 2  Sigmoidectomy – 4  LAR/APR – 7  Subtotal colectomy – 2 |
| (Klos et al., 2016) | SR: 6 (50)  PC:21 (67) | SR: mean 51 (SE 4)  PC: mean 56 (SE 3) | CD: 10  UC: 14  IBD-U: 3 | SR: Median 25 (range 19-44)  PC: Median 27 (range 17-68) | SR: Median 24 (range 9-30)  PC: Median 20 (range 0-44) | NA | NA | SR: Dysplasia – 0  Cancer – 6 (100)  PC: Dysplasia – 0  Cancer – 21 (100) | Proctectomy - 6  Proctocolectomy - 21 |
| (Maser et al., 2013) | 42 (56) | NA | CD: 75 (100) | Median 35 (range 10-95) | Median 11 (range 0-38) | Ileal only: 8  Ileocolonic: 40  Colonic only: 27 | NA | Dysplasia – 11 (15)  Cancer – 64 (85) | ‘Segmental resection’ – 54  Subtotal colectomy - 21 |
| (Lindberg et al., 2006) | SR: 8 (36)  PC: NA | SR: Median 44 (range 14-78)  SR and later PC: Median 33 (range 15-71)  PC: Median 42 (range 24-81) | CD: 0  UC: 51 (100) | Whole cohort: Median 28 (range 3-80) | SR: Median 13 (range 0-39)  SR and later PC: Median 8 (range 1-36)  PC: Median 13 (range 0-47) | NA | NA | SR: Dysplasia – 3 (14)  Cancer – 4 (18)  PC: Dysplasia – 6 (21)  Cancer – 2 (7) | Right Hemi – 1  Left hemi – 1  Sigmoidectomy – 1  Proctectomy – 1  Subtotal colectomy – 18 |
| Total N=4181  SR: 3810  PC: 371 | SR: 62%  PC: 82% | - | CD: 1989 (31)  UC: 3952 (61)  IBD-U: 359 (6)  IBD & cholangitis: 152 (2) | - | - | - | - | SR: Neoplasia 32-100%  PC: Neoplasia 28-100% | ‘Segmental resection’ – 1515  Right hemi – 975  Subtotal colectomy – 703  Proctectomy – 420  Sigmoidectomy – 174  Left hemi – 15 |

**Supplementary Table 2.** Demographics of the patients included in the systematic review. SR= segmental resection; PC= Proctocolectomy; IRA= ileorectal anastomosis; IPAA=ileal-pouch-anal anastomosis; DR=diverted rectum; CD=Crohn’s Disease; UC= Ulcerative Colitis; NA=Not Available.

| **Study ID** | **Disease duration at colectomy (years)** | **Length of Follow-up** | **Total metachronous lesions reported** | | **Time to recurrence** | **Stage of recurrent lesions** | |
| --- | --- | --- | --- | --- | --- | --- | --- |
|  |  |  | **Cancer (%)** | **Dysplasia (%)** | **Cancer** | **Cancer** | **Dysplasia** |
| Derks et al., 2023 | SR: Median 19 (IQR 13 -31) | Median 27 months (IQR 7 – 69) | 7/139 (5.0)* | 35/139 (25.2) | Median 27.5 months (IQR 14.0-46.0) | NS | IND/LGD – 26  HGD – 9 |
| Frontali et al., 2020 | NS | Median 40 months (range 1-600) | 3/72 (4.2) | 3/72 (4.2) | Median 1.58 years (range 0.2 -13.2 years) | NS | NS |
| Krugliak Cleveland et al., 2019 | Median 20.5 (range 5–46) | Median 17 months (range 3-228) | 0/17 | 4/17 (23.5) | - | 0 | LGD – 4 |
| Khan et al., 2017 | SR: Median 7 | Median 9 years | 0/25** | NS | - | Metastatic – 1 (liver) | NS |
| Klos et al., 2016 | SR: Median 24 (range 9-30) | Median 2.7 years (IQR 1.3 -10.1) | 2/6 (33.3) | NS | <2 years | Metastatic – 2 | NS |
| Maser et al., 2013 | Median 11 (range 0-38) | Mean 5.3 years; median 2.9 years (range 0-38yrs) | 22/75 (29.3) | 4/75 (5.3) | Median 3 years (range 1 – 38 years) | Localised – 20  Regional – 3  Metastatic – 2  (25 total recurrences, 3 synchronous) | LGD – 2  HGD – 2 |
| Lindberg et al., 2006 | SR: Median 13 (range 0-39)  SR and later PC: Median 8 (range 1-36) | ns | 0/22 | 3/22 (13.6) | - | 0 | LGD - 3 |
| **Total** | **-** | **-** |  |  | **-** | **Localised – 20 (65%)**  **Regional – 3 (10%)**  **Metastatic – 5 (16%)**  **Unknown – 3 (10%)** | **IND/LGD – 35 (71%)**  **HGD – 11 (22%)**  **Unknown – 3 (6%)** |

**Supplmentary Table 3.** Metachronous cancer and dysplasia (diagnosed > 6 months after colorectal resection) recurrences reported by the studies and the associated time to recurrence reported by the studies. *Segmental and subtotal colectomy patients**No intraluminal lesions were identified; One unknown, likely CRC-origin liver metachronous lesion was reported. SR = segmental resection; NS=not stated; LGD=Low Grade Dysplasia; HGD=High Grade Dysplasia.

| **Study ID** | **Co-morbidities**  **(ASA/Charlson)** | **Length of Stay**  **(days)** | **Complications**  **(****Clavien-Dindo, CD)** | | **Readmissions** | | **Completion surgery at end of follow-up**  **(%)** | **Indications for re-operation** |
| --- | --- | --- | --- | --- | --- | --- | --- | --- |
|  |  |  | **SR** | **PC** | **<30 days** | **>30 days** |  |  |
| Frontali et al., 2020 | ns | SR: Mean 10.9 (range 1 - 31) | CD <III: 7  CD >III: 10 | - | ns | ns | 22/72 (31)  *24 re-operations, 2 had further SR | Refractory colitis- 14/72  Cancer – 3/72  Dysplasia – 3/72  Stenosis – 1/72  Other – 3/72 |
| Krugliak Cleveland et al., 2019 | ns | ns | ns | - | ns | ns | 2/17 (12) | Dysplasia – 2/17 |
| Khan et al., 2017 | SR: ASA0-1: 8%  ASA2: 20%  ASA3+: 72%  PC: ASA0-1: 27%  ASA2: 21%  ASA3+: 53% | SR: Median 7  PC: Median 9 | CD <III: 0  CD >III: 0 | CD <III: 1  CD >III: 3 | SR: 0  PC: 4/34 | SR: 3/25  PC: 5/34 | ns | - |
| Lindberg et al., 2006 | ns | ns | ns | ns | ns | ns | 10/22 (45) | Refractory colitis – 6/22  Dysplasia – 3/22  Other – 1/22 |
| **Total** | **-** | **-** | **-** | **-** | **-** | **-** | **-** | **Refractory colitis – 20 (55.6%)**  **Cancer – 3 (8.3%)**  **Dysplasia 8 (22.2%)**  **Stenosis – 1 (2.8%)**  **Other – 4 (11.1%)** |

**Supplementary Table 4.** Surgical outcomes reported by the studies. SR=segmental resection; PC=proctocolectomy; ASA=American Society of Anaesthesiologists; ns=not stated.

| **Study ID** | **Co-morbidities**  **(ASA/Charlson)** | **Age at colectomy** | **Follow-up** | **Cohort size** | | **Deaths** | | |
| --- | --- | --- | --- | --- | --- | --- | --- | --- |
|  |  |  |  | **SR** | **PC** | **30-day** | **End of follow-up** | **Causes** |
| Frontali et al., 2020 | ns | Mean 57 (s.d. 17, range 14 - 84) | Median 40 months (range 1-600 months) | 72 | - | SR: 3 | SR: 3 | Intercurrent Illness – 3 |
| Maser et al., 2013 | ns | ns | Mean 5.3 years; median 2.9 years (range 0-38yrs) | 75 | - | ns | SR: 14 | Cancer - 7; Intercurrent illness - 2; Unknown - 5 |
| Khan et al., 2017 | SR: ASA0-1: 8%  ASA2: 20%  ASA3+: 72%  PC: ASA0-1: 27%  ASA2: 21%  ASA3+: 53% | SR: Median 73  PC: Median 61.7 | Median 9 years | 25 | 34 | SR: 0  PC: 0 | SR: 1  PC: 2 | SR: Cancer - 1  PC: Cancer - 2 |
| Lindberg et al., 2006 | ns | SR: Median 44 (range 14-78)  SR and later PC: Median 33 (range 15-71)  PC: Median 42 (range 24-81) | ns | 22 | 29 | ns | SR: 4  PC: 5 | SR: Cancer - 1  Intercurrent Illness - 3  PC: Intercurrent Illness - 5 |

**Supplementary Table 5.** Incidence of deaths as reported by the studies, and the causes. SR=segmental resection; PC=proctocolectomy; ASA=American Society of Anaesthesiologists; ns=not stated.

| **Study ID** | **Pre-op disease severity** | **Length of Follow-up** | **Disease relapse** | | **Severity of flare** | **No. on medical therapy at end of follow-up** | |
| --- | --- | --- | --- | --- | --- | --- | --- |
|  |  |  | **Colitis or Proctitis (%)** | **Pouchitis (%)** |  | **Colitis/Proctitis (%)** | **Pouchitis(%)** |
| Frontali et al., 2020 | Mayo 0 – 7  Mayo 1 – 21  Mayo 2 – 14  Mayo 3 – 7  Unk – 23 | Median 40 months (range 1-600 months) | 34/72 (47)* | - | Mild: 0  Mod/severe: 14  Unknown: 20 | 25/72(35) | - |
| Krugliak Cleveland et al., 2019 | ns | Median 17 months (range 3-228) | 0/17 | - | - | - | - |
| Khan et al., 2017 | SR/PC:  Froslie 0 – 15/8  Froslie 1 – 4/12  Froslie 2 – 6/14 | Median 9 years | 21/25 (84) | 9/34(26) | SR: Mild: 17  Mod/severe: 4  PC: Unknown: 9 | 21/25(84) | 9/34(26) |
| Lindberg et al., 2006 | ns | ns | 6/22 (27) | - | Mild: 0  Mod/severe: 6 | - | - |

**Supplementary Table 6.** IBD flare and Pouchitis incidence post-surgery at end of follow-up. *Study by Frontali et al. comprised of 51% patients undergoing segmental resection for non-neoplastic indications, including colitis. ns=not stated; SR=segmental resection; PC=proctocolectomy.

**References**

1. Kabir M, Fofaria R, Arebi N, Bassett P, Tozer PJ, Hart AL, et al. Systematic review with meta-analysis: IBD-associated colonic dysplasia prognosis in the videoendoscopic era (1990 to present). 2020 p. 5–19.

2. Laine L, Kaltenbach T, Barkun A, McQuaid KR, Subramanian V, Soetikno R. SCENIC international consensus statement on surveillance and management of dysplasia in inflammatory bowel disease. Gastroenterology. 2015;148(3).

3. Heuthorst L, Harbech H, Snijder HJ, Mookhoek A, D’haens GR, Everine Vermeire S´, et al. Increased Proportion of Colorectal Cancer in Patients With Ulcerative Colitis Undergoing Surgery in the Netherlands. 2022 [cited 2023 Jun 11]; Available from: https://doi.org/10.14309/ajg.0000000000002099

4. Gearhart SL, Nathan H, Pawlik TM, Wick E, Efron J, Shore AD. Outcomes from IBD-associated and non-IBD-associated colorectal cancer: A Surveillance Epidemiology and end results medicare study. Dis Colon Rectum. 2012;55(3).

5. Birch RJ, Burr N, Subramanian V, Tiernan JP, Hull MA, Finan P, et al. Inflammatory Bowel Disease-Associated Colorectal Cancer Epidemiology and Outcomes: An English Population-Based Study. Am J Gastroenterol [Internet]. 2022 Nov 1 [cited 2022 Nov 21];117(11):1858–70. Available from: https://journals.lww.com/ajg/Fulltext/2022/11000/Inflammatory_Bowel_Disease_Associated_Colorectal.28.aspx

6. Page MJ, Moher D, Bossuyt PM, Boutron I, Hoffmann TC, Mulrow CD, et al. PRISMA 2020 explanation and elaboration: updated guidance and exemplars for reporting systematic reviews. BMJ [Internet]. 2021 Mar 29 [cited 2022 Mar 31];372. Available from: https://pubmed.ncbi.nlm.nih.gov/33781993/

7. Critical Appraisal Tools | JBI [Internet]. [cited 2023 Mar 30]. Available from: https://jbi.global/critical-appraisal-tools

8. Bogach J, Pond G, Eskicioglu C, Simunovic M, Seow H. Extent of Surgical Resection in Inflammatory Bowel Disease Associated Colorectal Cancer: a Population-Based Study. J Gastrointest Surg [Internet]. 2021 Oct 1 [cited 2022 Jun 7];25(10):2610–8. Available from: https://pubmed.ncbi.nlm.nih.gov/33559097/

9. Klos CL, Safar B, Wise PE, Hunt SR, Mutch MG, Birnbaum EH, et al. Impaired outcome colitis-associated rectal cancer versus sporadic cancer. J Surg Res [Internet]. 2016 Jul 1 [cited 2022 Jun 20];204(1):123–9. Available from: https://pubmed.ncbi.nlm.nih.gov/27451878/

10. Krugliak Cleveland N, Ollech JE, Colman RJ, Rodriquez D, Hirsch A, Cohen RD, et al. Efficacy and Follow-up of Segmental or Subtotal Colectomy in Patients With Colitis-Associated Neoplasia. Clinical Gastroenterology and Hepatology [Internet]. 2019 Jan 1 [cited 2022 Jun 20];17(1):205–6. Available from: http://www.cghjournal.org/article/S1542356518304671/fulltext

11. Khan N, Cole E, Shah Y, Paulson EC. Segmental resection is a safe oncological alternative to total proctocolectomy in elderly patients with ulcerative colitis and malignancy. Colorectal Dis [Internet]. 2017 Dec 1 [cited 2022 Jun 20];19(12):1108–16. Available from: https://pubmed.ncbi.nlm.nih.gov/28498617/

12. Derks ME, te Groen M, Peters CP, Dijkstra G, de Vries AC, Romkens TE, et al. Endoscopic and surgical treatment outcomes of colitis-associated advanced colorectal neoplasia: a multicenter cohort study. Int J Surg [Internet]. 2023 Jun 8 [cited 2023 Aug 20];109(7). Available from: https://pubmed.ncbi.nlm.nih.gov/37300890/

13. Lindberg J, Stenling R, Palmqvist R, Rutegård J. Surgery for neoplastic changes in ulcerative colitis - Can limited resection be justified? Outcome for patients who underwent limited surgery. Colorectal Disease [Internet]. 2006 Sep 1 [cited 2022 Jun 20];8(7):551–6. Available from: https://onlinelibrary.wiley.com/doi/full/10.1111/j.1463-1318.2006.00997.x

14. Maser EA, Sachar DB, Kruse D, Harpaz N, Ullman T, Bauer JJ. High Rates of Metachronous Colon Cancer or Dysplasia After Segmental Resection or Subtotal Colectomy in Crohn’s Colitis. Inflamm Bowel Dis [Internet]. 2013 Aug;19(9):1827–32. Available from: www.ibdjournal.org

15. Frontali A, Cohen L, Bridoux V, Myrelid P, Sica G, Poggioli G, et al. Segmental Colectomy for Ulcerative Colitis: Is There a Place in Selected Patients Without Active Colitis? An International Multicentric Retrospective Study in 72 Patients. J Crohns Colitis [Internet]. 2020 Dec 1 [cited 2022 Jun 20];14(12):1687–92. Available from: https://pubmed.ncbi.nlm.nih.gov/32498084/

16. Kiran RP, Nisar PJ, Goldblum JR, Fazio VW, Remzi FH, Shen B, et al. Dysplasia associated with Crohn’s colitis: Segmental colectomy or more extended resection? Ann Surg. 2012 Aug;256(2):221–6.

17. Choi CHR, Rutter MD, Askari A, Lee GH, Warusavitarne J, Moorghen M, et al. Forty-year analysis of colonoscopic surveillance program for neoplasia in ulcerative colitis: An updated overview. Vol. 110, American Journal of Gastroenterology. 2015.

18. Krugliak Cleveland N, Colman RJ, Rodriquez D, Hirsch A, Cohen RD, Hanauer SB, et al. Surveillance of IBD Using High Definition Colonoscopes Does Not Miss  Adenocarcinoma in Patients with Low-grade Dysplasia. Inflamm Bowel Dis. 2016 Mar;22(3):631–7.

19. Derikx LAAP, Nissen LHC, Smits LJT, Shen B, Hoentjen F. Risk of Neoplasia After Colectomy in Patients With Inflammatory Bowel Disease: A Systematic Review and Meta-analysis. Clinical Gastroenterology and Hepatology. 2016;14(6).

20. Kabir M, Thomas-Gibson S, Hart AL, Tozer PJ, Faiz O, Warusavitarne J, et al. Management of inflammatory bowel disease associated colonic dysplasia: factors predictive of patient choice and satisfaction. Colorectal Disease. 2021;23(4).
